# Low-Level Laser and Antimicrobial Photodynamic Therapy Reduce Peri-implantitis–related Microorganisms Grown In Vitro

**DOI:** 10.1055/s-0041-1731926

**Published:** 2021-10-01

**Authors:** Marcelo H. Tonin, Fabiano C. Brites, José R. Mariano, Karina M. S. Freitas, Mariana A. L. Ortiz, Samira Salmeron

**Affiliations:** 1 Department of Implantology, Ingá University Center, Maringá, Paraná, Brazil; 2 Department of Implantology, Unieuro University Center, Brasília, Brazil; 3 Department of Orthodontics, Ingá University Center, Maringá, Paraná, Brazil; 4 Department of Microbiology, Ingá University Center, Maringá, Paraná, Brazil; 5 Department of Periodontics and Implant Dentistry, Ingá University Center, Maringá, Brazil

**Keywords:** decontamination, lasers, photodynamic therapy, biofilm, *Staphylococcus aureus*

## Abstract

**Objective** Currently, dental implants are a predictable treatment option for oral rehabilitation; however, complications such as peri-implant diseases are increasing every day. Thus, the aim of this study was to verify the efficacy, *in vitro* , of two protocols against cultures of periodontal biofilm and *Staphylococcus aureus* .

**Material and Methods** Petri dishes for each of the following groups were used: control groups (C)—plates inoculated with periodontal biofilm (C.B; *n* = 4) or *S. aureus* (C.SA; *n* = 4) without any treatment; laser groups—plates inoculated with periodontal biofilm (low-level laser therapy [LLLT].B; *n* = 4) or *S. aureus* (LLLT.SA; *n* = 4) and treated with LLLT (660 nm, 30 mW, 50 J/cm ^2^ , and 47 seconds); antimicrobial photodynamic therapy groups (aPDT)—plates inoculated with periodontal biofilm (aPDT.B; *n* = 4) or *S. aureus* (aPDT.SA; *n* = 4) and treated with aPDT (red laser 660 nm, 30 mW, 50 J/cm ^2^ , 47 seconds + toluidine blue O (TBO) 100 µg/mL, and 1 minute). After treatments were performed, the contents of all plates were diluted and seeded for counting colony-forming units (CFUs).

**Statistical Analysis** Results were analyzed with one-way analysis of variance (ANOVA), Tukey’s test, comparison of percentages, and independent *t* -tests with a 5% significance level.

**Results** Both treatments, LLLT and aPDT, significantly reduced the number of CFUs for the two types of culture, LLLT.B (3.69 × 10 ^6^ ± 0.20), aPDT.B (2.79 × 10 ^6^ ± 0.13), LLLT.SA (4.10 × 10 ^6^ ± 0.12), and aPDT.SA (3.23 × 10 ^6^ ± 0.10) when compared with control groups C.B (5.18 × 10 ^6^ ± 0.43) and C.SA (5.81 × 10 ^6^ ± 0.16; *p* = 0.000). When treatment groups were compared separately, there was also a statistically significant difference ( *p* = 0.000). None of the protocols were able to eliminate cultured microorganisms.

**Conclusion** The LLLT and aPDT protocols effectively reduced cultures of periodontal biofilm and *S. aureus in vitro* , with the superiority of aPDT.

## Introduction

Dental implants are currently a highly predictable treatment option for cases of oral rehabilitation, with high success rates 1 and survival. 2 However, with the increase in the number of implants installed and the “aging” of these implants in the oral cavity, the number of complications also increases, for example, the peri-implant diseases. 3

Peri-implant diseases, according to the recent classification, include peri-implant mucositis and peri-implantitis. 4 Peri-implant mucositis is defined as an inflammatory lesion that affects the mucosa around the implants without loss of supporting bone. 5 It is a reversible condition, once the biofilm is eliminated, characterized by bleeding on probing and visual signs of inflammation. 5 Peri-implantitis presents a much more critical clinical situation. It is a pathological condition associated with a biofilm that involves the tissues around the implants, characterized by inflammation of the peri-implant mucosa, and subsequent and progressive bone loss. 6

Recent evidence indicates that peri-implant mucositis is a precursor of peri-implantitis in the same way that gingivitis is of the periodontitis. 7 Just as the biofilm is also considered the main etiological factor for peri-implant and periodontal diseases. 4 7 8 Studies show that the peri-implantitis microbiota is similar to the periodontitis, 9 10 11 including species of the red complex such as *Porphyromonas gingivalis, Tannerella forsythia* , and *Treponema denticola* . 12 However, there may be differences in its composition 10 13 with the identification of microorganisms not commonly found in periodontitis like *Staphylococcus aureus, Staphylococcus epidermidis* , and *Candida spp* . 14

The peri-implantitis microbiota is quite complex and heterogeneous, and its characterization remains incomplete. 6 Its microbiome is characterized by a great diversity of microorganisms composed of aerobic gram-positive, anaerobic gram-negative, and fusiform pathogens. 15 Persson and Renvert 10 identified a different bacterial profile in peri-implantitis, including *P. gingivalis, S. aureus, Staphylococcus anaerobius, Streptococcus intermedius, Streptococcus mitis, T. forsythia* , and *Treponema socranskii* . Sahrmann et al, 15 in a systematic review and meta-analysis, found a high prevalence of *Aggregatibacter actinomycetemcomitans* and *Prevotella intermedia* in biofilms of implants with peri-implantitis compared with healthy implants. Rates of microorganisms identified in peri-implantitis that are less common in periodontitis, such as *S. aureus* and *S. epidermidis* , are reported in the literature. 10 Infection by *S. aureus* , in particular, may be important in the development of peri-implantitis, 10 16 17 since, during the formation of the biofilm, this microorganism acts as a primary colonizer, creating favorable conditions for late bacteria adherence and colonization. 10

Thus, biofilm removal becomes essential in cases of peri-implantitis 17 18 ; the prevention and treatment of this condition are even critical for the long-term stability of implants. 18 It is suggested that the successful treatment of peri-implantitis is based on biofilm removal associated with the implant surface decontamination. In this context, several protocols for peri-implantitis treatment and implant surface decontamination are seen in literature. 16 Chemical and mechanical methods have been proposed, but until now, no protocol is considered ideal for peri-implantitis treatment. 19

The use of lasers and antimicrobial photodynamic therapy (aPDT) for decontamination of the titanium implant surfaces has been studied with very promising results. 19 20 The great advantage of laser therapies is the absence of consequences seen in other methods such as local irritation and the development of bacterial resistance. 16 However, the effect of the laser alone as a decontaminant of implant surfaces is controversial topic in literature. Some studies report good results 19 21 and others report no differences between laser and conventional treatments. 22 23 24

In the last decades, the association of low-level lasers with photosensitizers has also been used to reduce or eliminate bacteria, known as aPDT. However, the large number of questions about the topic and the wide range of protocols described in the literature still do not assure the use of aPDT as an adjunct therapy in the treatment of peri-implantitis. Therefore, the present study aimed to verify the *in vitro* efficacy of two protocols, using low-level laser therapy (LLLT) and aPDT, against cultures of periodontal biofilm and *S. aureus* .

## Material and Methods

### Microorganisms and Growth Conditions

The sample of periodontal biofilm was frozen and obtained from the sample of the Laboratory of Microbiology at Ingá University Center, Uningá. 20 It is a subgingival biofilm collected from a patient diagnosed with periodontitis. The *S. aureus* used was a standard strain (ATCC 25923) from the bacteria collection from the same laboratory at Ingá University Center, Uningá, which was frozen in Brain Heart Infusion (BHI) broth (Kasvi, São José dos Pinhais, Brazil) with 15% glycerol. For use, the strain was thawed and transferred to Petri dishes containing culture medium and then incubated at 37°C for 24 hours for reactivation.

For the preparation of the inoculum, both the periodontal biofilm and the *S. aureus* strain were standardized by transferring colonies of these microorganisms to a tube with saline until turbidity corresponding to the 0.5 MacFarland scale, which corresponds to approximately 1.5 × 10 ^8^ colony-forming units (CFU)/mL.

### Distribution of Experimental Groups

Petri dishes containing 5 mL of standardized inoculums were distributed into the following groups, according to the culture of microorganisms: periodontal biofilm (B) or *S. aureus* (SA):

- Control groups: plates inoculated with periodontal biofilm (C.B; *n* = 4) or *S. aureus* (C.SA; *n* = 4) without any treatment.
- Low-level laser therapy groups: plates inoculated with periodontal biofilm (LLLT.B; *n* = 4) or *S. aureus* (LLLT.SA; *n* = 4) and treated with low-level laser therapy.
- Antimicrobial photodynamic therapy groups: plates inoculated with periodontal biofilm (aPDT.B; *n* = 4) or *S. aureus* (aPDT.SA; *n* = 4) and treated with aPDT.

Standard microbiological tests were also performed to ensure the sterility of the culture medium.

### Photosensitizer Solution and Light Source

The dye used was toluidine blue O (TBO; Sigma-Aldrich Brazil, São Paulo, Brazil), at a concentration of 100 µg/mL, diluted in distilled water. The solution was prepared and handled under restricted light conditions. The laser used was the LLLT diode, InGaAlP (Whitening Lase II, DMC Equipment Ltda, São Carlos, Brazil). The irradiation parameters are described in Table 1 .

**Table 1:** Laser parameters

**Type of laser**	**Diode—InGaAlP**
Emission mode	Scan
Wavelength (nm)	660
Power (mW)	30
Time (s)	47
Energy density (J/cm ^2^ )	50
Tip area (cm ^2^ )	0.0028
Energy (J)	0.14

### Microorganism Culture Treatments

#### Low-level Laser Therapy

**Fig. 1:**
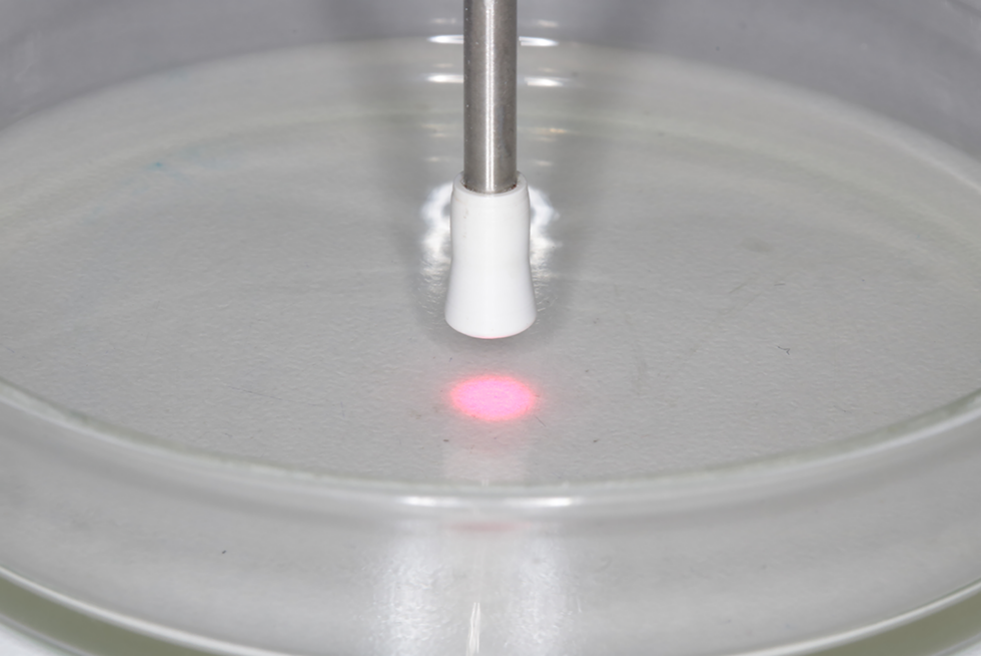
Petri dish containing the microorganisms and being irradiated with low-level laser (red) according to the protocol.

The standardized inoculums of 5 mL were transferred to sterile Petri dishes according to the groups LLLT.B and LLLT.SA. The plates were irradiated with laser for 47 seconds, in scan mode, by a single operator, with tip/plate distance of 1 cm, and according to the parameters described in Table 1 , inside the laminar flow chamber ( **Fig. 1** ).

#### Antimicrobial Photodynamic Therapy

**Fig. 2 (A):**
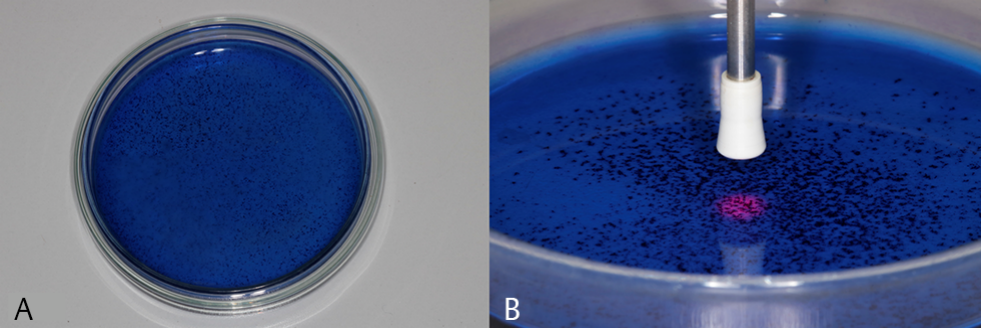
Preirradiation period. Petri dish containing the microorganisms and TBO applied for 1 minute before irradiation. ( **B** ) Irradiation. Petri dish containing the microorganisms immerged in TBO and being irradiated with low-level laser (red) according to the protocol. TBO, toluidine blue O.

The standardized inoculums of 5mL were transferred to sterile Petri dishes according to the groups aPDT.B and aPDT.SA. Then, 5 mL of the TBO solution was placed on the plates. After 1 minute, laser irradiation was performed for 47 seconds, in scan mode, by a single operator, with tip/plate distance of 1 cm, according to the parameters described in Table 1 ( **Fig. 2** ).

### Colony-Forming Units Counting

After treatments, the material from all plates, including the control groups, was diluted to a concentration of 10 ^−3^ . Then, 100 µL of these dilutions were transferred to blood agar plates, in duplicate, stored in a CO _2_ PVC - polyvinyl chloride jar (Permution, Curitiba, Brazil) to guarantee the microaerophilic condition (candle jar technique, 5% CO _2_ ), and placed in an incubator for 48 hours at 37°C, allowing colonies to grow. After this period, CFUs were counted with the naked eye, by an experienced and calibrated examiner.

### Statistical Analysis

The normality of the data was assessed by the Shapiro–Wilk test. The one-way analysis of variance (ANOVA) and post hoc Tukey’s tests were used to compare the CFUs among the experimental groups. For the comparison of CFUs between treatments separately, the independent *t* -test was used. And for the comparison of CFUs reduction between test groups was performed the comparison of percentages. These tests were performed with Statistica software (Statistica for Windows version 10.0, Statsoft, Tulsa, Oklahoma, United States). The data were considered significant for *p* < 0.05.

## Results

**Fig. 3:**
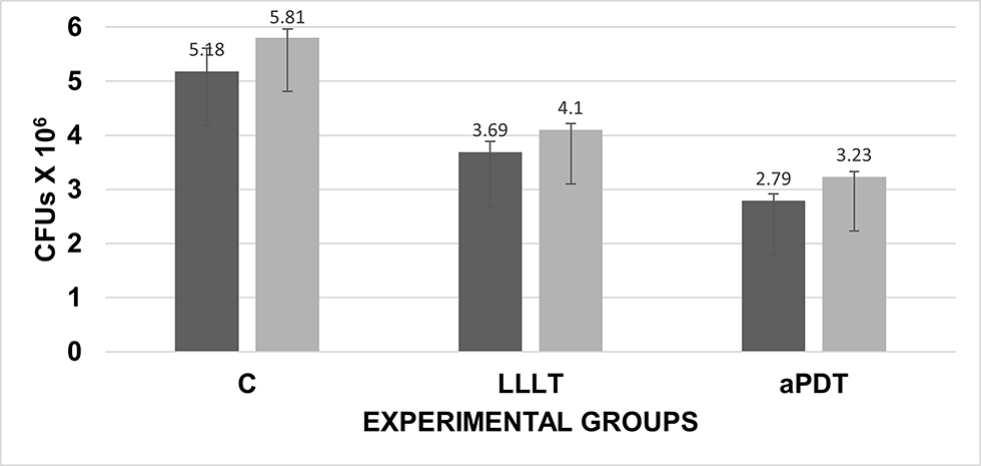
Number of colony-forming units (CFUs) on the experimental groups. Control (C), low-level laser therapy (LLLT), and antimicrobial photodynamic therapy (aPDT). Periodontal biofilm (dark bars); *Staphylococcus aureus* (light bars). Error bars indicate standard deviation (one-way ANOVA and post hoc Tukey’s test). ANOVA, analysis of variance.

There was a statistically significant difference in the number of CFUs among the groups evaluated, both for the periodontal biofilm and *S. aureus* . The treatments, LLLT and aPDT, significantly reduced the number of CFUs of both cultures compared with controls ( *p* = 0.000; one-way ANOVA and post hoc Tukey’s test; **Fig. 3** ). The antimicrobial action of both treatments was similar against periodontal biofilm and *S. aureus* with reduction of 28.77% and 29.44% CFUs, respectively, using LLLT treatment ( *p* = 0.488; comparison of percentages) and reduction of 46.14% CFUs of periodontal biofilm and 44.41% CFUs of *S. aureus* , using aPDT treatment ( *p* = 0.472; comparison of percentages; Table 2 ).

**Table 2:** Values of reductions (P%) in the number of CFUs considering control groups representing 100% of microorganisms’ growth

Groups	CFUs	Reduction (P%)	*p* ^*^
LLLT-B	71.23% (3.69 × 10 ^6^ )	28.77% (1.49 × 10 ^6^ )	0.488
LLLT-SA	70.56% (4.10 × 10 ^6^ )	29.44% (1.71 × 10 ^6^ )	
aPDT-B	53.86% (2.79 × 10 ^6^ )	46.14% (2.39 × 10 ^6^ )	0.472
aPDT-SA	55.59% (3.23 × 10 ^6^ )	44.41% (2.58 × 10 ^6^ )	
Abbreviations: aPDT, antimicrobial photodynamic therapy; B, periodontal biofilm; CFU, colony-forming unit; LLLT, low-level laser therapy; SA, *Staphylococcus aureus* . ^*^ Statistically significant for *p* < 0.05 (comparison of percentages).			

When treatments were compared separately, there was statistically significant difference ( *p* = 0.000; independent *t* -test). A lesser number of CFUs for periodontal biofilm and *S. aureus* were observed after treatment with aPDT than with LLLT ( Table 3 ). However, none of the treatments eliminated the microorganisms.

**Table 3:** Means and standard deviations of the CFUs of the LLLT and aPDT groups compared separately according to microorganisms’ culture

Groups	CFUs	*p* ^*^
LLLT-B	3.69 × 10 ^6^ (0.20)	0.000*
aPDT-B	2.79 × 10 ^6^ (0.13)	
LLLT-SA	4.10 × 10 ^6^ (0.12)	0.000*
aPDT-SA	3.23 × 10 ^6^ (0.10)	
Abbreviations: aPDT, antimicrobial photodynamic therapy; B, periodontal biofilm; CFU, colony-forming unit; LLLT, low-level laser therapy; SA, *Staphylococcus aureus* . ^*^ Statistically significant for *p* < 0.05 (independent *t* -test).		

## Discussion

The present study tested the LLLT and aPDT against periodontal biofilm and *S. aureus in vitro,* and the results showed good efficacy of both protocols.

As previously stated, peri-implantitis is a complex clinical condition that presents a variable and heterogeneous microbiota. 6 From this perspective, finding which method is most effective against the microorganisms involved in the clinical condition of peri-implantitis is an important step in establishing protocols for treating the disease.

The literature shows that *in vitro* models of biofilm containing several species can serve as useful tools in studying various polymicrobial infections. 25 Thus, this study used periodontal biofilm previously collected from a volunteer with periodontitis. 20 This subgingival biofilm sample was selected due to the similarities with the microbiota presented in cases of peri-implantitis. 9 10 11 The morphotinturial analysis, performed immediately after collection and before freezing, revealed the presence of gram-positive and gram-negative microorganisms 20 ; however, there was no characterization of this biofilm, with the identification of microorganisms, due to the need for more complex analyzes that demand higher costs and specific equipment.

The selected *S. aureus* strain is standard (ATCC 25923). *S. aureus* was chosen because it requires special attention throughout the peri-implantitis process due to its ability to firmly adhere to the titanium surface 26 and its possible participation on the biofilm formation as a primary colonizer, creating favorable conditions for the adhesion of bacteria of late colonization. 10

In this study, two protocols of antimicrobial effect using LLLT and aPDT were analyzed. The LLLT protocol was adapted from Salmeron et al 19 for decontamination of titanium discs contaminated by oral biofilm *in situ* and evaluated *in vivo* . The adaptation was related to the fluency parameters and exposure time changed due to the equipment used that had these parameters prefixed according to protocol selection. The equipment was the same used by this research group 20 with this irradiation protocol for aPDT. In the previous study, 19 the fluency and exposure time were lower, 45 J/cm ^2^ and 30 seconds, respectively, and similar results were found, with good potential for decontamination of the discs using the LLLT.

The present results also suggest good efficacy of the LLLT in the parameters used, with a significant reduction in the number of CFUs in the periodontal biofilm and *S. aureus* groups compared with control groups. De Sousa et al 27 , used the same wavelength and power (660 nm; 30 mW) and also obtained a reduction in the growth of *S. aureus* with the difference that they used lower fluences than used in this study.

Different effects can be seen in the action of the laser on gram-positive and gram-negative bacteria, 27 probably due to structural differences that can affect the penetration of laser irradiation and mediate differences in susceptibility. 27 It can be related and explain partially the similar results in reduction of CFUs presented in this study for periodontal biofilm and *S. aureus* , since there was gram-positive microorganisms in periodontal biofilm too.

The studies still present controversial data regarding the antimicrobial action of the laser, considering the difficulty in standardizing the protocols due to the number of parameters and the type of laser used. For this reason, in the last decades, the association of LLLT with photosensitizers has also been used to reduce or eliminate bacteria, in the called aPDT. 19 20 28 In this study, the aPDT protocol was the same used by Batalha et al 20 and similar results were obtained. These authors used the same sample of periodontal biofilm to contaminate dental implants *in vitro* and have a significant reduction in the number of CFUs compared with control group.

The effects of aPDT involve several parameters, not only referring to the laser but the dye as well. The interaction of these photosensitizer dyes with the laser wavelength is essential for the correct functioning of the mechanism of action of aPDT. 29 Researchers have preferred TBO for interacting better with lipopolysaccharides from gram-negative bacteria even without a light source, 30 according to some studies reporting the use of the dye alone. 19 28 Besides, TBO has an intense absorption in the region of 620 to 660 nm 29 which justifies selecting the wavelength used in this study. The preirradiation time of 1 minute was also defined based on literature. 19 20 28

Although the reduction in the number of CFUs for both cultures with both protocols was quite satisfactory, it has not yet been sufficient to eliminate microorganisms. This indicates that the association of physical and chemical methods, as suggested by Cai et al, 16 can be used for decontamination of dental implant surfaces. Another interesting finding was the significant difference between the aPDT results compared with LLLT results, separately for both cultures. The aPDT reduced the number of CFUs more than the LLLT for periodontal biofilm and *S. aureus* , with better antimicrobial action. And the efficacy of both treatments was similar against periodontal biofilm and *S. aureus* .

## Limitations

It is noted that the use of LLLT and aPDT is effective in combating the growth of several microorganisms, especially the aPDT that presented a superior antimicrobial action, suggesting that it can be an adjunct therapy option in the treatment of peri-implant diseases. However, protocols and therapies that employ the laser have limitations, that is, there are no specific forms of application, emphasizing the need for further studies to improve the preexisting protocols, since these therapies are very promising.

## Conclusion

Based on the results obtained and considering the limitations of this *in vitro* study, it was possible to conclude that the protocols of LLLT and aPDT used were effective in reducing cultures of periodontal biofilm and *S. aureus* , with the superiority of antimicrobial photodynamic therapy.
